# Evolution and Comparative Analysis of Sheep Reference Genomes: From Fragmented Assemblies to Telomere-to-Telomere Genomics

**DOI:** 10.3390/biology15060465

**Published:** 2026-03-13

**Authors:** Dan Yue, Ying Lu, Yuqing Chong, Jiao Wu, Zhendong Gao, Ruoshan Ma, Keyu Li, Weidong Deng, Bo Wang

**Affiliations:** 1 Yunnan Provincial Key Laboratory of Animal Nutrition and Feed, Faculty of Animal Science and Technology, Yunnan Agricultural University, Kunming 650201, China; 2 Department of Animal Science, Yuxi Agriculture Vocation-Technical College, Yuxi 653106, China

**Keywords:** sheep, reference genome, T2T assembly, genome evolution, multi-omics integration

## Abstract

The reference genome serves as a fundamental resource for sheep genetic research and molecular breeding, and iterative improvements in assembly quality have directly driven advances in these fields. A systematic literature review was conducted by retrieving relevant studies from major scientific databases using predefined keywords related to sheep reference genomes and genome assembly technologies, followed by structured screening and comparative analysis of eligible publications. This review systematically summarizes the developmental trajectory of the sheep reference genome from early fragmented assemblies to telomere-to-telomere (T2T) genome assembly approaches. Different genome versions are comparatively analyzed from multiple perspectives, including assembly strategies, quality metrics, and functional annotation. Importantly, we propose a genome-resolution-driven analytical framework that explicitly links successive improvements in reference genome completeness with paradigm shifts in sheep genetic analysis, ranging from marker-based studies to structural variation- and multi-omics-enabled trait dissection. Particular emphasis is placed on the potential applications and biological research value of T2T genomes in assembly methodologies and multi-omics integrative analyses. Furthermore, the practical significance of different reference genome versions in genetic dissection, trait mapping, and breeding applications is reviewed, and future directions for sheep genome research are discussed. This review provides both a systematic synthesis and a forward-looking conceptual roadmap for advancing sheep molecular genetics and precision breeding in the T2T genomics era.

## Simple Summary

High-quality reference genomes are essential tools for understanding the genetic makeup of organisms and for applying this knowledge in agriculture and biology. In sheep, reference genomes have undergone continuous improvement over the past decade, evolving from early fragmented versions to nearly complete genomes that span entire chromosomes from end to end. However, many researchers and breeders are still unclear about how these improvements have changed what can be studied and achieved in sheep genetics. In this review, we summarize the development of sheep reference genomes from early incomplete assemblies to recent near-complete genome versions. We compare different genome versions in terms of their completeness, accuracy, and practical usefulness, and explain how better genome quality has enabled new types of genetic analyses. Our analysis shows that improvements in genome resolution have made it possible to study complex genetic features that were previously inaccessible, including repetitive regions and large structural changes in the genome. Overall, this work provides a clear overview of how advances in genome technology have transformed sheep genetic research and highlights how high-quality reference genomes can support future studies, livestock improvement, and more precise breeding strategies, benefiting both science and agricultural production.

## 1. Introduction

Sheep (*Ovis aries*), one of the earliest domesticated livestock species, are generally believed to have been domesticated approximately 10,000–11,000 years ago [1,2]. Through long-term artificial selection and natural adaptation, sheep have gradually diversified into a wide range of breeds with highly heterogeneous phenotypes, becoming an indispensable component of the global livestock industry [3]. From an economic perspective, sheep provide essential agricultural products such as meat, wool, and milk, and occupy an important position in global agricultural production systems [4]. China is one of the world’s major sheep-producing countries and plays a critical role in global sheep production, contributing to food security, the economic development of pastoral regions, and the maintenance of ecosystem stability [5]. However, sheep production systems are globally distributed across diverse ecological zones, including European temperate regions, African arid and semi-arid systems, Middle Eastern drylands, South American pastoral systems, and Eurasian continental steppe environments [6]. Understanding genome evolution and adaptation across these diverse production contexts is essential for constructing globally representative genomic resources. In addition, sheep serve as an important livestock model for studying adaptive evolution and the genetic architecture of complex traits [7]. Research on high-altitude hypoxia adaptation, high fecundity, and unique phenotypic traits in sheep has provided valuable resources for elucidating adaptive evolutionary processes and the genetic regulatory networks underlying economically important traits [8].

As a “roadmap” for genomic research, the completeness and accuracy of a reference genome directly determine the reliability of studies on genetic variation detection, gene mapping, and functional annotation [9,10]. In livestock genomics, continuous improvements in reference genome quality have driven paradigm shifts in research approaches—from early gene cloning and candidate gene studies, to chromosome-level genome-wide association studies (GWAS), and further to systematic analyses of structural variation and complex gene regulatory networks enabled by highly contiguous genome assemblies [11]. This progression has expanded both the depth and resolution of genetic dissection for complex traits [12]. Importantly, the practical utility of these advances in breeding programs is mediated through their impact on commercial genotyping platforms. The quality of the reference genome directly determines the accuracy of SNP chip design, including probe placement precision, genome-wide marker distribution uniformity, and the cross-breed portability of SNP panels. Improvements in assembly contiguity and annotation completeness reduce probe misplacement and enhance polymorphism validation rates across diverse populations, thereby indirectly but substantially increasing the practical value of SNP-based genomic selection in sheep breeding [13,14].

The development of the sheep reference genome has been closely linked to advances in sequencing technologies. From first-generation Sanger sequencing, to second-generation Illumina short-read sequencing, and further to the integrated application of third-generation PacBio HiFi sequencing, Oxford Nanopore Technologies (ONT) ultra-long reads, and Hi-C chromatin conformation capture, the quality of sheep genome assemblies has progressively advanced from fragmented drafts toward highly contiguous, low-gap, and ultimately T2T assemblies [15,16]. These technological advances have laid a solid foundation for resolving previously inaccessible complex repetitive regions and structural variations [17].

Since the International Sheep Genomics Consortium (ISGC) released the first sheep reference genome, Oar_v1.0, in 2009, sheep genome assemblies have undergone multiple major upgrades. This initial assembly was constructed using a donor individual from the Texel breed, a specialized meat breed originating from the Netherlands [18,19]. The ISGC, a global collaborative framework established to coordinate sheep genome sequencing efforts, played a foundational role in the early phases of sheep genomics by pooling resources and expertise from multiple countries. Subsequent versions (Oar_v2.0 to Oar_v3.1) represented iterative improvements based on the same Texel-derived genomic framework, incorporating additional short-read sequencing data to refine contiguity and gene annotation, but retained the same underlying breed background. Early versions (Oar_v1.0–Oar_v3.1) provided an initial genomic framework but were characterized by extensive gaps and fragmented regions [20,21]. Subsequent versions (Oar_v4.0 and Oar_v5.0) incorporated long-read sequencing and Hi-C technologies to achieve chromosome-level assemblies, becoming the mainstream reference genomes for GWAS and molecular marker development [22,23]. These improved assemblies, while representing significant technological advances, remained largely derived from the original Texel lineage, meaning that the underlying breed-specific genomic architecture remained consistent across Oar_v1.0 through Oar_v5.0 [19]. Nevertheless, these versions still exhibit substantial limitations in highly repetitive regions, including centromeres, telomeres, and sex chromosomes [24,25]. In recent years, with the maturation of ultra-long-read sequencing and chromatin conformation capture technologies, research efforts have increasingly focused on constructing sheep T2T genomes [26,27]. Notably, T2T should be distinguished from conventional chromosome-level assemblies: chromosome-scale scaffolding can still retain unresolved gaps and collapsed repeats, particularly in centromeres, telomeres, segmental duplications, and parts of sex chromosomes. By contrast, T2T assemblies aim to provide end-to-end continuity and a more faithful representation of repeat architecture, thereby expanding the callable genome for complex SV/CNV discovery and reducing reference-related mapping bias across breeds [27,28,29]. Exploratory studies, reported in preprints and intermediate-stage publications, have reduced long-standing unresolved complex repetitive regions present in traditional reference genomes, providing a new technical foundation for multi-omics integrative analyses and the genetic dissection of complex traits [30,31].

Guided by this gap, the central objective of this review is to frame sheep genomics through the lens of reference genome resolution and to clarify how improvements in assembly completeness reshape both discovery and application. Specifically, we aim to: (i) trace the trajectory of sheep reference genomes from fragmented drafts to emerging T2T assemblies; (ii) compare major genome versions in terms of assembly strategies, continuity and completeness metrics, and annotation performance; (iii) articulate a resolution-driven analytical framework that explains the field’s transition from SNP- and interval-based analyses to structure-aware variation discovery and regulatory interpretation; and (iv) evaluate how T2T genomes may enable more reliable multi-omics integration, cross-breed variant interpretation, and breeding-relevant genotyping and selection strategies. At the population level, this improved resolution reduces reference bias and improves SV/CNV discovery across breeds, while at the breeding level it supports more accurate marker placement, haplotype definition, and genotype imputation underpinning genomic selection. Collectively, this synthesis provides a conceptual roadmap for sheep genomics in the T2T era.

## 2. Evolution of the Sheep Reference Genome

**Figure 1:**
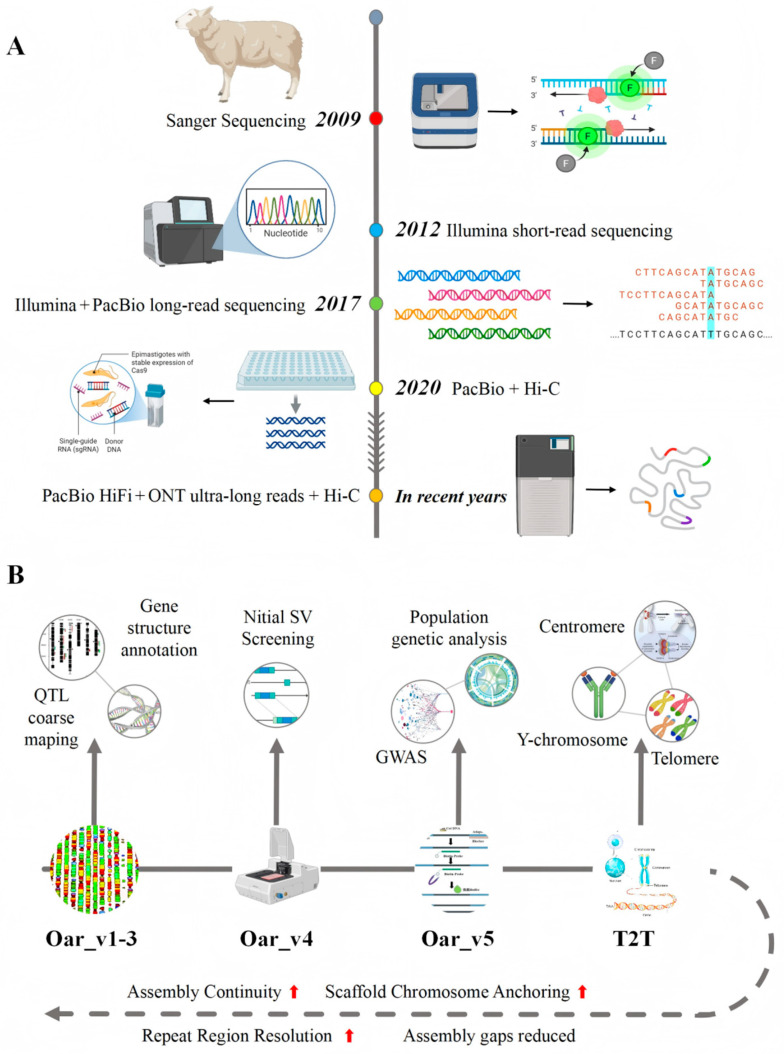
Technological evolution and improvement in resolution capacity of sheep reference genomes from early fragmented assemblies to T2T assemblies. (**A**) Timeline illustrating key milestones in sheep reference genome development, from the initial Oar_v1.0 (2009) to recent T2T assemblies, showing the progressive integration of advanced sequencing technologies (Sanger, Illumina, PacBio, Hi-C, ONT ultra-long) and the corresponding improvements in assembly continuity and completeness. (**B**) Quantitative improvements in key assembly metrics across successive genome versions, including contig N50, scaffold N50, BUSCO completeness, and gap number reduction. The transition from kilobase-scale contigs (Oar_v1.0) to megabase-scale (Oar_v4.0/v5.0) and ultimately to chromosome-level T2T assemblies illustrates the co-evolution of sequencing technology and assembly quality. Together, panels (**A**,**B**) demonstrate how technological advances (**A**) directly drive measurable improvements in assembly quality metrics (**B**).

The development of the sheep reference genome has been closely intertwined with iterative advances in sequencing technologies [32]. From the release of the first official assembly, Oar_v1.0 (Texel sheep), in 2009 to recent exploratory efforts and milestone progress toward T2T genome assemblies (Figure 1A), substantial improvements have been achieved in assembly continuity, annotation completeness, and the resolution of complex genomic regions [31,32,33]. Overall, the trajectory from early fragmented drafts to chromosome-level assemblies and emerging T2T resources reflects the stepwise integration of longer reads, improved scaffolding strategies, and more mature assembly and validation workflows.

### 2.1. Construction of Initial Reference Genomes

Sheep genomics research began with the whole-genome sequencing project launched by the ISGC in 2007. The ISGC, a global collaborative framework established to coordinate sheep genome sequencing efforts, played a foundational role in the early phases of sheep genomics by pooling resources and expertise from multiple countries [34]. Based on Sanger dideoxy sequencing technology, the first sheep reference genome assembly, Oar_v1.0 (https://www.ncbi.nlm.nih.gov/datasets/genome/GCA_000005525.1/ (accessed on 16 February 2026)), was constructed, with a contig N50 of approximately 11 kb, a genome coverage of ~90%, and a total assembled length of ~2.66 Gb, providing an initial framework of the sheep genome [9]. This version annotated approximately 20,000 protein-coding genes, laying a foundation for comparative genomics studies. However, due to the inherent limitations of Sanger sequencing in read length and cost for high-throughput assembly, this early assembly contained numerous gaps and fragmented regions. In particular, repetitive sequence–rich regions such as centromeres, telomeres, and parts of intergenic regions were poorly resolved, and a substantial proportion of tandem repeats remained unassembled [20,21].

Subsequently, through the gradual integration of Illumina short-read data and optimization of De Bruijn graph–based assembly algorithms, updated versions including Oar_v2.0 (2010), Oar_v3.0 (2012), and Oar_v3.1 (2014) were released. Oar_v2.0 incorporated medium- to high-coverage Illumina HiSeq short reads, increasing the contig N50 to the tens-of-kilobases range and reducing the number of assembly gaps. Oar_v3.1 further integrated RNA-seq transcriptomic data to refine gene structure annotation, improving the completeness of protein-coding gene annotation, with the contig N50 stably exceeding 50 kb and overall assembly quality substantially enhanced relative to earlier versions [10,35].

### 2.2. Quality Improvements in Mid- to Late-Stage Reference Genomes

With the maturation of second-generation sequencing technologies and the introduction of third-generation long-read sequencing, sheep reference genome assembly entered a high-quality phase. The release of Oar_v4.0 in 2017 marked the first large-scale integration of long-read data generated by the PacBio RS II platform, combined with optical mapping for auxiliary error correction. This stage reflects a shift from a primarily centralized consortium model toward a distributed but interconnected international research network. This approach increased the contig N50 to the megabase scale, reduced gap numbers, and improved genome continuity, particularly in intergenic regions and regions containing moderately repetitive sequences [36,37].

The subsequent release of Oar_v5.0 in 2020 further incorporated Hi-C chromatin conformation capture technology, enabling chromosome-level anchoring of 26 autosomes and the sex chromosomes. As a result, the scaffold N50 increased to the hundreds-of-megabases scale, and both the completeness and consistency of gene annotation were further improved relative to the previous version [23,38]. By integrating multi-tissue transcriptomic datasets—including liver, muscle, and skin—and incorporating epigenomic information, Oar_v5.0 systematically optimized the annotation of repetitive sequences and non-coding RNAs, enriching annotations of lncRNAs and miRNAs and correcting selected gene structure models [39,40]. Crucially, the integration of transcriptomic and epigenomic datasets was greatly facilitated by global initiatives such as the Functional Annotation of Animal Genomes (FAANG) consortium, which established community standards for data production and annotation, thereby strengthening cross-species comparability and annotation consistency [39,41]. As a result, Oar_v5.0 has been widely adopted as the primary reference genome in sheep genomics studies in sheep genetics research during this stage [42,43].

Despite these advances, mid- to late-stage reference genomes still exhibited assembly gaps in highly complex genomic regions, including centromeric satellite repeats, telomeric and subtelomeric regions, and copy number variation–enriched regions, with complete resolution of centromeric regions remaining particularly limited [24]. These unresolved regions restricted the systematic identification of functional genes and genetic variants. For example, studies on gastrointestinal nematode resistance in sheep have noted that genetic variation analysis of regulatory regions for certain candidate genes is constrained by their proximity to telomeric or subtelomeric regions in existing reference genomes [44]. Moreover, the incomplete assembly of the Y chromosome during this stage limited comprehensive multi-omics analyses of male-specific genomic regions and associated traits [45].

### 2.3. Breakthrough Progress in T2T Reference Genomes

In recent years, with advances in ultra-long-read sequencing and chromatin conformation capture technologies, research efforts have increasingly focused on constructing T2T sheep reference genomes, achieving milestone progress in reducing long-standing unresolved complex regions present in traditional reference assemblies. These initiatives are often embedded within broader high-contiguity genome programs and international genome infrastructure projects, involving multidisciplinary teams that integrate livestock geneticists, structural genomics experts, bioinformaticians, and high-performance computing centers [46,47]. In addition to experimental design, these projects depend heavily on high-performance computing infrastructure capable of processing ultra-large sequencing datasets and executing computationally intensive assembly algorithms. Such T2T assembly studies typically use high-quality domesticated sheep individuals as donors and integrate PacBio HiFi sequencing, ONT ultra-long reads, and Hi-C data, complemented by optical mapping and other validation datasets [31]. Compared with traditional reference genomes, these approaches reduce unresolved complex repetitive regions, substantially enhance assembly continuity and overall completeness, and achieve meaningful improvements in resolving specific telomeric and centromeric regions [24,30].

At the level of functional annotation, T2T assemblies provide a more continuous genomic framework for refining annotations in previously intractable repetitive regions [41], and also improve the characterization of centromeric repeat architectures and their chromosomal distributions [26,48]. The iterative evolution of sheep reference genomes reflects the co-evolution of sequencing technology innovation and assembly algorithm optimization [49], setting the stage for systematic cross-version comparisons and resolution-aware analyses in subsequent sections.

## 3. Systematic Comparison of Different Versions of Sheep Reference Genomes

The iterative evolution of sheep reference genomes essentially reflects the co-evolution of sequencing technology innovation and assembly algorithm optimization [49], as well as the progressive maturation of international institutional collaboration frameworks. From the ISGC-led foundational phase to later distributed global research networks and functional annotation consortia, organizational evolution has paralleled technological advancement and has shaped data accessibility, annotation standards, and cross-breed comparative research. Differences among genome versions in assembly continuity, annotation completeness, and application suitability directly determine the depth and breadth of sheep genomics research [11]. Beyond a simple technical comparison, this section introduces a resolution-based evaluation framework to systematically assess how successive improvements in reference genome quality reshape analytical capabilities, research questions, and biological interpretability in sheep genomics. Through a systematic comparison of key assembly metrics, functional annotation, and application scenarios (Table 1), we delineate a clear transition from framework-level genome utilization toward gap-free, function-oriented genomic analyses, providing practical guidance for selecting appropriate reference genomes under different research objectives [50,51,52].

### 3.1. Comparison of Assembly Quality and Functional Annotation Completeness

Core quality metrics of sheep reference genomes have continuously improved in parallel with advances in sequencing technologies (Figure 1B) [16]. In terms of assembly continuity, the Sanger sequencing–based Oar_v1.0 exhibited low contiguity, with a contig N50 only at the kilobase scale [21]. The introduction of Illumina short-read data improved contig continuity in Oar_v3.1, although its ability to resolve repetitive sequences remained constrained by short-read length limitations [18]. The incorporation of PacBio long-read sequencing in Oar_v4.0 enabled the contig N50 to reach the megabase scale for the first time, improving continuity in intergenic regions and moderately repetitive sequences [55]. Subsequent integration of Hi-C technology in Oar_v5.0 achieved chromosome-level scaffold anchoring, enhancing large-scale structural integrity and chromosomal organization [23,38]. Alongside these improvements, overall assembly completeness also increased markedly, with mid- to late-stage reference genomes such as Oar_v5.0 achieving high BUSCO completeness, reflecting improved coverage of conserved core gene sets relative to early fragmented assemblies [56,57].

Despite substantial gains in contiguity and completeness, traditional reference genomes still exhibit limited resolution of highly repetitive regions, including centromeric satellite repeats, telomeric and subtelomeric regions, and copy number variation–enriched loci [58]. Although mid- to late-stage assemblies substantially reduced gap numbers, unresolved fragments persist in centromeric and subtelomeric regions [25]. In recent years, T2T assembly strategies have demonstrated clear advantages in further filling these complex regions. By integrating PacBio HiFi sequencing, ONT ultra-long reads, and Hi-C data, T2T assemblies enhance continuous coverage of highly repetitive regions and provide new technical pathways for more comprehensive assessment of sheep genome completeness [26,27].

**Figure 2:**
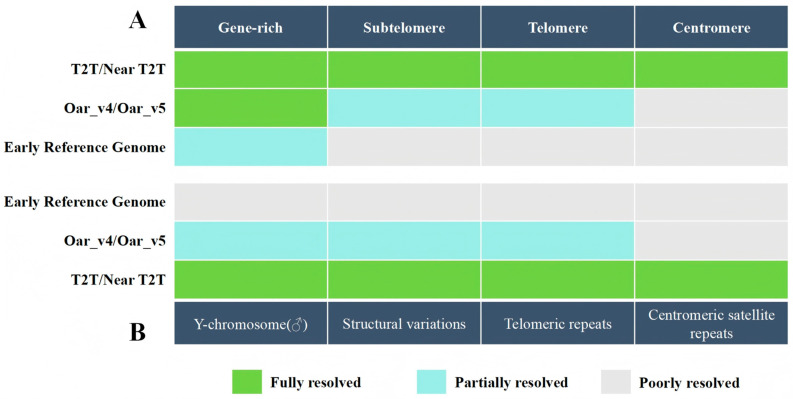
Comparison of assembly completeness across complex genomic regions in different reference versions. (**A**) Assembly completeness across functional and structural regions of autosomes. The assembly performance of three genome versions was compared across gene-rich regions, subtelomeric regions, telomeric regions, and centromeric regions. (**B**) Assembly quality in the Y chromosome and repetitive structural regions. The three genome versions were further compared in the Y chromosome (♂), structural variations, telomeric repeats, and centromeric satellite repeats.

In addition to improvements in continuity and completeness, assembly accuracy at the single-nucleotide level has also progressed across reference genome versions (Figure 2). Early assemblies were constrained by limited sequencing depth and error-correction strategies, resulting in relatively high base-level error rates [20,59]. The introduction of multi-platform sequencing and integrated correction pipelines in mid- to late-stage reference genomes improved base accuracy [36]. T2T assembly studies that leverage high-fidelity long-read data, particularly PacBio HiFi reads, further enhance nucleotide-level precision, providing a more reliable reference framework for high-confidence detection of single-nucleotide variants and small-scale structural variations [16].

As reference genome quality has improved, the completeness and accuracy of functional annotation in sheep have also steadily advanced (Table 1). Early genome versions relied primarily on homology-based annotation approaches, which successfully identified major protein-coding genes but exhibited limitations in gene structure accuracy, annotation depth, and systematic identification of non-coding RNA elements [60]. By integrating multi-tissue transcriptomic datasets, Oar_v4.0 and Oar_v5.0 substantially optimized gene structure prediction and functional annotation workflows, improving protein-coding gene annotation accuracy and enriching annotations of non-coding RNAs such as lncRNAs and miRNAs [22]. Nevertheless, functional annotation of repetitive sequences remains incomplete, particularly for centromeric satellite repeats and Y chromosome–specific repetitive elements [24]. Recent T2T-based studies further integrate multi-omics data—including single-cell transcriptomics, epigenomics, and Hi-C chromatin conformation information—providing new opportunities to refine annotation in these regions and enabling the identification of previously under-annotated protein-coding genes and non-coding RNAs, some of which exhibit tissue-specific expression patterns [61,62].

### 3.2. Comparison of Application Scenarios and Limitations

Differences in application scenarios among sheep reference genome versions are largely determined by assembly continuity, the ability to resolve complex repetitive regions, and the completeness of functional annotation [32]. Due to high fragmentation and numerous gaps, early reference genome versions were primarily suitable for gene cloning, genetic marker development, and preliminary QTL mapping of economically important traits, but exhibited clear limitations in the fine-scale dissection of complex quantitative traits and the systematic identification of structural variations [45]. Chromosome-level assemblies (e.g., Oar_v4.0 and Oar_v5.0) substantially improved coordinate reliability and chromosomal organization, thereby supporting mainstream GWAS and population-scale analyses; however, highly repetitive regions such as centromeres, telomeres, and parts of the Y chromosome remain incompletely resolved, which continues to constrain studies targeting SV/CNV-enriched loci, sex chromosome–specific variation, and chromosomal structural evolution [63]. Recent T2T and near-T2T assemblies further reduce unresolved complex repeats and improve continuity in these difficult regions, offering a higher-resolution reference framework for methodological development and for resolving key genomic segments, although current resources remain limited in breed coverage and are not yet positioned to fully replace mainstream references in routine large-scale population analyses and breeding practice [27,31,51].

## 4. Technological Breakthroughs of Sheep T2T Genomes and New Insights into Basic Biology

The completion of the T2T sheep genome represents a substantial technical advance in achieving gapless and highly continuous genome assemblies in livestock [28,30]. This breakthrough not only provides a more precise reference framework for sheep genomics research but also creates the necessary conditions for an in-depth resolution of the molecular mechanisms underlying their genetic foundation, evolutionary adaptation, and important economic traits [64].

### 4.1. Technological Innovations in Sheep T2T Genome Assembly

The construction of T2T-sheep1.0 was based on an integrated assembly strategy that synergistically combines multiple sequencing platforms. Unlike early genome projects driven primarily by a single international consortium, contemporary T2T assembly efforts reflect a mature collaborative ecosystem characterized by shared computational pipelines, cross-institutional validation strategies, and open-access data dissemination frameworks. Therefore, the feasibility of constructing T2T-level sheep reference genomes is not solely determined by sequencing platforms but also by access to advanced computational infrastructure, including large-memory HPC clusters, parallelized workflow management systems, and long-term data storage facilities. This infrastructural dimension represents a critical yet often under-discussed component of next-generation livestock genomics [27,65]. Systematic optimization was achieved at multiple levels, including data type configuration, workflow design, and the resolution of complex genomic regions, providing an important technical demonstration for advancing livestock genomes from highly contiguous assemblies toward gap-free reference genomes [28,66].

At the data acquisition level, the study adopted a combinatorial strategy integrating PacBio HiFi sequencing, ONT ultra-long reads, and Hi-C data, thereby fully exploiting the complementary strengths of different sequencing technologies in accuracy, long-range continuity, and chromosomal spatial positioning [15]. High-accuracy HiFi reads provide robust support for single-nucleotide–level assembly precision and gene annotation, while ONT ultra-long reads exhibit clear advantages in spanning complex regions such as satellite repeats and transposon-rich regions [16,62]. Hi-C data further enable chromosomal-scale ordering, orientation, and correction of misassemblies by leveraging spatial chromatin interaction information [67]. This multi-platform integration strategy effectively mitigates assembly fragmentation and misjoining caused by repetitive sequences [68].

At the raw data level, PacBio HiFi sequencing and ONT ultra-long read platforms can generate datasets reaching hundreds of terabytes, particularly when deep coverage is required for resolving highly repetitive regions. The storage, transfer, and management of such data necessitate high-capacity distributed storage systems and stable data management pipelines. From a computational perspective, de novo assembly of T2T genomes typically requires high-performance computing (HPC) clusters equipped with terabyte-scale memory (often 1–2 TB RAM per node or higher) to handle large assembly graphs and repeat-rich regions. Long-read error correction, consensus polishing, haplotype resolution, and repeat separation rely on computationally intensive graph-based algorithms and iterative correction pipelines [69,70]. These processes may require multiple rounds of alignment, polishing, and structural refinement, substantially increasing computational time and resource consumption. Notably, this study made significant advances in resolving highly repetitive regions, especially putative centromeric regions. By integrating repeat unit features, DNA methylation signals, and homology-based alignment, a framework was developed to classify and annotate and to evaluate assembly consistency, improving the reliability of assemblies across putative centromeric regions. Compared to existing sheep genomes, this approach enhanced the completeness of putative centromeric regions and their flanking sequences, providing a solid foundation for future chromosome function and evolution studies [26]. However, sequence-resolved satellite arrays alone do not establish functional centromeres; functional validation typically requires chromosomal localization (e.g., FISH with satellite probes) and/or mapping of centromere-specific proteins (e.g., CENP-A ChIP-seq) in relevant sheep cell types.

### 4.2. New Insights into Sheep Genome Structure and Function Revealed by T2T Genomes

Leveraging the gap-free sequences of T2T-sheep1.0, studies have systematically characterized the sequence composition and structural features of putative centromeric regions in sheep. By applying an integrated identification framework originally developed for human centromeres [26], researchers have revealed that the core regions of sheep centromeres are primarily composed of tandem arrays of specific satellite repeat units, with pronounced differences in repeat copy number and regional length among chromosomes. Whether centromere size variation (or satellite copy number) affects segregation fidelity in sheep remains unclear and warrants cross-breed comparisons using the T2T reference. However, these inferences are based on sequence architecture and associated epigenomic patterns, and do not by themselves demonstrate that the newly resolved satellite arrays correspond to active, CENP-A–defined functional centromeres.

The completeness of T2T genomes has substantially enhanced the detection of genetic variation in sheep, particularly structural variants. Whole-genome comparisons with traditional reference assemblies have revealed numerous insertions, deletions, and copy number variations that were previously difficult to identify accurately [71]. Functional annotation and pathway summaries in the original reports highlighted genes overlapping these SVs that are involved in immune response, metabolism, and reproduction; however, the statistical significance and multiple-testing criteria of these enrichments were not consistently reported across studies [72]. However, whether these SVs are breed-specific has not been consistently assessed across datasets, and the inferred regulatory impacts are largely based on genomic overlap with coding or putative regulatory regions rather than direct experimental validation. Therefore, these conclusions should be interpreted as hypothesis-generating, and mechanistic links between specific SVs and phenotypes remain to be validated experimentally.

Building upon the gap-free sequence of T2T-sheep1.0, extensive gene model refinement and supplementation have been performed in telomeric regions, centromere-adjacent regions, and other highly repetitive genomic areas. Among the newly annotated protein-coding genes, a subset exhibits tissue-specific or differential expression in organs related to environmental adaptation, immune defense, and reproduction, providing new candidate resources for downstream functional studies [73]. However, the authenticity and expression of these newly annotated genes would benefit from independent transcriptomic support and targeted validation (e.g., RT-qPCR across multiple tissues). Nevertheless, the biological significance of these genes requires further confirmation through genetic manipulation and population-level validation [74]. In addition, several candidate genes potentially involved in chromosome stability and cell division regulation have been identified in telomeric and centromere-proximal regions, offering new insights into meiotic progression and chromosome segregation mechanisms. The specific functions of these genes and their roles in trait formation, however, remain to be elucidated through functional genetic experiments.

Notably, sheep represent a particularly informative model for T2T-based genomic research among livestock species. Compared with cattle and goats, sheep exhibit pronounced breed stratification, extensive adaptation to diverse and extreme environments, and complex chromosomal structural variation shaped by domestication and artificial selection. These features amplify the biological significance of resolving previously inaccessible genomic regions, such as centromeres, telomeres, and the Y chromosome [63]. As a result, T2T assemblies in sheep not only improve technical completeness but also provide unique opportunities to investigate how structural variation, repeat architecture, and chromosomal organization contribute to phenotypic diversity, environmental adaptation, and economically important traits.

## 5. Advances in Genetic Dissection and Applications Driven by Sheep Reference Genomes

Building on the resolution-based comparison above, we next discuss how reference improvements translate into advances in population evolution, physiological function, and production traits. The realization of the T2T sheep genome represents an important technical advance in basic genomic research and provides a high-quality reference framework that supports genetic dissection and molecular breeding applications (Table 2) [30,31]. It extends the focus of genomic research from the acquisition of the sequences themselves to the in-depth interpretation of associations between genetic variations and phenotypes, as well as the substantial empowerment of practical breeding efforts [12].

### 5.1. Population Evolution and Population-Genetic Inference Enabled by Improved References

Progressive improvements in sheep reference genomes have substantially strengthened population genetics and evolutionary inference by reducing reference bias and increasing the callable proportion of the genome, particularly in repeat-rich and structurally complex regions. Early fragmented assemblies were adequate for SNP-based summaries in unique regions, yet ambiguous read placement around long repeats, segmental duplications, and CNV/SV hotspots often inflated missingness and distorted allele-frequency estimates, thereby limiting robust cross-population comparisons and demographic inference [45]. Chromosome-level references (e.g., Oar_v4.0 and Oar_v5.0) improved coordinate consistency and long-range genomic organization, enabling more reliable population-scale analyses based on GWAS, LD patterns, ROH, and genome-wide selection scans across breeds and ecological types [23,38]. However, unresolved centromeric and telomeric repeats and incomplete sex chromosome representation continued to constrain the interpretation of selection signals and structural evolution in these regions [63].

Near-T2T and T2T assemblies further mitigate these limitations by correcting potential structural misassemblies, resolving long repetitive tracts, and improving breakpoint representation for large SVs and CNVs, which enhances SV discovery, genotyping accuracy, and the comparability of population datasets [26,45]. This is particularly important when adaptive divergence or domestication signals are enriched in structurally dynamic regions, where reference errors or collapsed repeats can otherwise generate spurious signals or mask true population differentiation. Moreover, more complete Y chromosome assemblies provide a necessary framework for evaluating male-specific variation, paternal lineage history, and sex-linked evolutionary dynamics, thereby extending population genetic analyses beyond autosomes to a genome-wide evolutionary perspective [26,45]. Together, these developments support a shift from SNP-centric population analyses toward structure-aware, resolution-matched evolutionary studies, and they provide the methodological basis for integrating multi-breed near-T2T resources and pan-genome directions in future population genomics of sheep [75].

### 5.2. Evolution of Reference Genomes and Enhanced Capacity for Trait Genetic Dissection

The continuous evolution of sheep reference genomes has substantially improved the capacity for genetic dissection of complex traits [76]. As assemblies have progressed from early fragmented references to highly contiguous and ultimately gap-free genomes, research paradigms have gradually shifted from simple “trait–marker associations” toward integrative frameworks linking traits to causal genes and underlying molecular mechanisms [77].

Early reference genomes primarily supported the mapping of simple Mendelian traits, exemplified by the identification of the *FecB* (*BMPR1B*) gene underlying prolificacy and associations between the *KRTAP* gene family and wool-related traits [78]. However, limited assembly continuity constrained analytical resolution, resulting in broad QTL intervals and making it difficult to resolve trait-associated signals located within repeat-rich or structurally complex genomic regions [79]. These early reference genome versions provided essential support for the construction of sheep genetic maps and the preliminary mapping of quantitative trait loci (QTLs) associated with economically important traits. Nevertheless, due to the intrinsic read-length limitations of short-read sequencing technologies, these early assemblies remained inadequate for spanning long repetitive elements and large structural variation regions, resulting in pronounced constraints on systematic structural variant analysis and the investigation of complex gene regulatory networks [45].

The release of Oar_v4.0 and Oar_v5.0 markedly improved the resolution of GWAS, enabling more precise genetic dissection of complex quantitative traits related to growth, meat quality, and disease resistance [43,80]. Numerous candidate genes and genetic variants involved in growth regulation, lipid metabolism, and immune responses have since been identified, providing valuable resources for downstream molecular breeding efforts [81]. The subsequent development of T2T-sheep1.0 further alleviated long-standing bottlenecks in resolving complex genomic regions, allowing genetic variation in previously inaccessible regions to be systematically incorporated into analytical frameworks and opening new possibilities for investigating breed-specific and adaptive traits [82]. In addition, T2T-based studies have corrected potential structural misassemblies present in existing sheep references, which helps reduce reference-driven artifacts in mapping and variant interpretation [26,45]. Moreover, improved assembly of the Y chromosome in T2T/near-T2T resources provides a more complete basis for dissecting male-specific sequences and advancing research on sex determination and reproduction-related genetic mechanisms in sheep [26,45].

### 5.3. Application Potential of T2T Genomes in Molecular Breeding

High-quality reference genomes constitute a critical foundation for the development of molecular breeding strategies [83]. With improvements in genome continuity and completeness, sheep breeding has gradually evolved from marker-assisted selection based on individual loci toward integrative selection approaches that leverage genome-wide information [84]. During the Oar_v5.0 stage, the development and application of high-density SNP arrays improved the accuracy of breeding value prediction and achieved favorable outcomes in the improvement of meat, wool, and reproductive traits [85]. Importantly, the quality of the underlying reference genome directly determines SNP probe positioning accuracy, genome-wide marker distribution uniformity, and the reliability of genotype calling. Early SNP chips designed based on fragmented assemblies (e.g., Oar_v1.0–Oar_v3.1) were constrained by incomplete contiguity and unresolved repetitive regions, resulting in uneven marker spacing and underrepresentation of telomeric, centromeric, and structurally complex regions. Misassembled or collapsed repetitive sequences could lead to ambiguous probe hybridization and reduced validation rates across genetically diverse breeds [86,87].The availability of chromosome-level assemblies (Oar_v4.0 and Oar_v5.0) improved physical coordinate precision and facilitated more rational SNP selection strategies, enabling more uniform genome coverage and improved probe specificity. Enhanced assembly accuracy also reduced false-positive polymorphism detection caused by misassembly artifacts, thereby increasing cross-population validation efficiency and improving the stability of genomic estimated breeding values (GEBVs) [88].

However, because traditional reference genomes incompletely resolve structural variants and copy number variation–enriched regions, certain genomic segments remain poorly represented in existing SNP arrays. The emergence of T2T assemblies provides an opportunity to redesign next-generation SNP chips by incorporating markers located within previously inaccessible repetitive or structurally complex regions and by accounting for breed-specific structural variation. Such improvements may enhance prediction accuracy for complex traits influenced by large structural variants or regulatory elements embedded in repetitive sequences [89]. The introduction of T2T genomes further expands this framework by enabling the integration of single-nucleotide variants, structural variants, and regulatory elements, thereby broadening both the genomic coverage and informational dimensions of breeding markers and selection targets [90].

In addition, T2T genomes provide more precise sequence references for gene-editing research, contributing to reduced off-target risks and improved reliability of genome-editing design [91]. Emerging evidence suggests that functional validation and targeted editing experiments based on high-quality genomic resources can deepen understanding of regulatory mechanisms underlying key economic traits [92]. Nevertheless, the large-scale application of such approaches in breeding programs will require continued evaluation of ethical considerations, biosafety, and long-term effects [93].

### 5.4. Contributions of T2T Genomes to Fundamental Biological Research

The completion of the T2T-sheep1.0 genome represents not only a leap in the quality of genomic maps but also a landmark for sheep as a model livestock species entering the “post-genomic era.” Beyond practical applications, T2T-sheep1.0 provides core support for fundamental biological research in sheep [30,31]. In studies of origin and domestication, chromosomal evolution, and functional genomics, the gapless genome enables systematic and in-depth analysis of previously inaccessible “dark matter” regions, such as telomeres, centromeres, and highly repetitive transposable element (TE) regions [94].

In the fields of chromosomal evolution and structural biology, T2T sequences have successfully conquered centromeres—the final “forbidden zone” of genome assembly [26]. Comparative genomic analysis indicates that transposable element-mediated chromosomal rearrangements (CAs) have played a central driving role in the evolution of sheep chromosomes. By precisely locating rearrangement breakpoints, researchers have revealed lineage-specific chromosomal fusion and fission events in sheep [48]. As the core of chromosome segregation, the internal satellite DNA within centromeres exhibits significant species-specificity. Research has found that the evolutionary rate of these sequences is much higher than the genomic average, suggesting that centromeres may harbor key potential biological functions in species differentiation and the maintenance of reproductive stability [95].

Concurrently, the T2T genome has completely captured various classes of transposable elements (TEs), providing foundational data for studying their roles in gene regulation and environmental adaptation [96]. In early and mid-stage genomic technologies, TEs—including LINEs, SINEs, and LTRs—were frequently misassembled or omitted [95]. However, T2T technology allows researchers to analyze how TEs function by remodeling chromatin accessibility or providing cis-regulatory elements. For instance, under extreme environmental stress, the activity of specific retrotransposons correlates positively with the expression of genes related to high-altitude adaptation and cold tolerance, indicating that TEs contribute substantially to shaping the phenotypic plasticity of complex traits in sheep [26,48].

At the levels of epigenetics and functional genomics, the gapless sequence provides an ideal template for whole-genome DNA methylation studies [48]. Researchers have discovered that the binding regions of centromere protein components (e.g., CENP-A) often correspond to “dips” in DNA methylation; this unique epigenetic pattern is crucial for maintaining the stability of meiosis. Furthermore, the T2T genome eliminates “hidden genes,” particularly multi-copy gene families located within repetitive regions. Through the precise measurement of telomeric repeats and the differentiation of highly similar paralogous genes, it is now possible to more accurately map quantitative trait nucleotides (QTNs) affecting traits such as meat yield, wool quality, and fecundity, greatly enhancing the precision and depth of sheep functional genomics research [42,97].

## 6. Conclusions

With the continuous advancement of sequencing technologies and assembly algorithms, sheep reference genomes have progressively evolved from early framework-level assemblies to chromosome-scale and, more recently, near T2T high-quality genomic resources. Improvements in assembly continuity, annotation completeness, and the resolution of complex repetitive regions across successive reference genome versions have substantially advanced sheep genomics research, particularly in structural variant identification, complex trait dissection, and multi-omics integrative analyses. Overall, early reference genomes played a foundational role in basic genetic research, mid- to late-stage chromosome-level assemblies have become the mainstream frameworks for population genetic analyses and molecular breeding, and T2T-based assembly strategies provide critical complements for resolving long-standing challenging regions such as centromeres, telomeres, and sex chromosomes.

Importantly, progressive improvements in reference genome resolution have driven a conceptual transition in sheep genomics, shifting analyses from marker- and interval-based approaches toward more function-oriented and structure-aware investigations. Building upon these advances, highly contiguous reference genomes have expanded our understanding of sheep chromosomal architecture, repeat sequence composition, and their evolutionary characteristics, while providing a more complete sequence context for identifying functional elements and structural variants. Although current studies demonstrate considerable potential in elucidating adaptive evolution and production-related traits, most findings remain primarily based on association analyses or limited breed representation, and both causal mechanisms and population-wide generalizability require further validation. While improvements in reference genome quality enhance annotation completeness, accurate functional interpretation ultimately depends on experimental characterization of individual gene functions. At present, many livestock gene annotations are still largely inferred from human orthologs and disease-associated databases, highlighting the need for more species-specific functional studies to strengthen functional genomics research in livestock species.

Looking forward, the development of high-quality pangenome resources across diverse sheep breeds, together with the cost-effective integration of T2T-level references and multi-omics data, is expected to facilitate the translation of genomic resources from descriptive assemblies toward mechanistic insights and precision breeding applications.

## Figures and Tables

**Table 1:** Systematic comparison of major sheep reference genome versions, assembly strategies, quality metrics, and application characteristics.

Index	Oar_v1.0 (2009) GCA_000298735.1	Oar_v3.1 (2014) GCA_000298735.1	Oar_v4.0 (2017) GCF_000298735.2	Oar_v5.0 (2020) GCF_002742125.1	T2T Assembly Exploration GCA_016772045.1	Exploration of Multi-Variety T2T/Near-T2T Assembly GCA_016772045.2
Publication Year	2009	2014	2017	2020	Recent Years	Recent Years
Core Sequencing Technologies	Sanger Sequencing	Sanger + Illumina short read sequencing	Illumina + PacBio long read long + optical spectrum	Illumina + PacBio + Hi-C	PacBio HiFi + ONT ultra-long read length + Hi-C	PacBio HiFi/ONT/Hi-C(Combined Application)
Contig N50	About 10 kb	50 kb	~1 Mb level	Not explicitly reported	>50 Mb (reported in individual studies)	From tens of Mb to over 100 Mb
Scaffold N50	About 2 Mb	Not systematically reported	Not explicitly reported	~260 Mb (chromosome level)	chromosome level	chromosome level
BUSCO Capture Rate	About 85–90%	Not systematically assessed (or About 85–90%)	90%	usually above 90%	usually above 90%	95%
Gap Quantity	~100,000	Significantly Less Than Oar_v1.0	Tens of thousands	Tens of thousands	significantly reduced/nearly gap-free	significantly less than the traditional reference genome
Genome integrity	About 90%	90%	93%	~95%	exhibits high integrity	95%
Single Base error rate	~10^−4^	~10^−4^–10^−5^	~10^−5^	~10^−5^	lower than traditional short-read assembly	order of magnitude ~10^−6^
Number of protein-coding genes	About 20,000	About 19,000–20,000	About 21,000–22,000	About 22,000	slightly higher than Oar_v5.0 (related to annotation strategy)	About 22,000–24,000
Core Features and Highlights	The first sheep reference genome, preliminarily outlining the overall genomic framework	Optimize gene annotation to support the development of basic genetic markers and preliminary QTL mapping	First large-scale introduction of long-read data improves genomic continuity	Achieving chromosome-level mapping is the mainstream reference genome for GWAS and structural variant studies	Exploratory implementation of telomere-telomere continuous assembly improves the resolution capability of complex repetitive regions such as telomeres and centromeres	Exploring T2T or near-T2T assembly strategies across different sheep breeds provides a technical foundation for pan-genome construction and breed-specific genetic analysis
References	[20]	[16,53]	[10,49]	[21,45,54]	[27]	[27]

**Table 2:** Impact of the evolution of sheep reference genomes on genetic analysis and downstream applications.

Reference Genome Stage	Representative Versions	NCBI Assembly Accession	Major Analytical Capabilities	Types of Genetic Variants Resolved	Typical Applications	Main Limitations
Early fragmented reference genomes	Oar_v1.0–Oar_v3.1	GCA_000298735.1	Gene localization and coarse-scale interval analysis	SNPs, small InDels	Mapping of Mendelian traits, preliminary QTL detection, marker development	Large QTL intervals; poor resolution of repetitive regions and structural variants
Chromosome-level reference genomes	Oar_v4.0	GCA_000298735.2	High-resolution GWAS and population genetic analyses	SNPs, partial CNVs and SVs	GWAS for growth, reproduction and disease resistance; high-density SNP chip design; genomic selection	Centromeric, telomeric and Y chromosome regions remain incompletely resolved
	Oar_v5.0	GCF_002742125.1				
Near T2T/T2T reference genomes	T2T-sheep1.0 and related assemblies	GCF_016772045.1	Gap-free genome-wide resolution	SNPs, SVs, CNVs and repetitive sequence variation	Fine mapping of complex traits, functional analysis of structural variants, studies of adaptive evolution	High sequencing cost; limited sample size and breed coverage
Multi-breed T2T/pan-genome direction	Multi-breed near-T2T assemblies	GCA_016772045.2	Resolution of breed-specific genetic variation	Core and variable genes	Comparative breed analysis, pan-genome construction, integration of resources for precision breeding	Still exploratory; lack of unified standards and pipelines

## Data Availability

No new data were created or analyzed in this study. Data sharing is not applicable to this article.
